# Functionalized 4-Hydroxy Coumarins: Novel Synthesis, Crystal Structure and DFT Calculations

**DOI:** 10.3390/molecules16010384

**Published:** 2011-01-07

**Authors:** Valentina Stefanou, Dimitris Matiadis, Georgia Melagraki, Antreas Afantitis, Giorgos Athanasellis, Olga Igglessi-Markopoulou, Vickie McKee, John Markopoulos

**Affiliations:** 1 Laboratory of Inorganic Chemistry, Department of Chemistry, University of Athens, Panepistimio-polis, Athens 15771, Greece; E-Mail: stef-valentin@windowslive.com (V.S.); 2 Laboratory of Organic Chemistry, School of Chemical Engineering, National Technical University of Athens, Zografou Campus, Athens 15773, Greece; E-Mails: dmatiadis@yahoo.gr (D.M.); georgiamelagraki@gmail.com (G.M.); afantitis@novamechanics.com (A.A.); g_athanasellis@yahoo.com (G.A.); ojmark@chemeng.ntua.gr (O.I.-M.); 3 Chemistry Department, University of Loughborough, Leicestershire, LE113TU, UK; E-Mail: v.mckee@lboro.ac.uk (V.M.)

**Keywords:** coumarins, *N*-hydrocysuccinimide ester, *C*-acylation, β,β΄-dicarbonyl system, cyclization, DFT

## Abstract

A novel short-step methodology for the synthesis in good yields of functionalized coumarins has been developed starting from an activated precursor, the *N-*hydroxysuccinimide ester of *O-*acetylsalicylic acid. The procedure is based on a tandem *C*-acylation-cyclization process under mild reaction conditions. The structure of 3-methoxycarbonyl-4-hydroxy coumarin has been established by X-ray diffraction analysis and its geometry was compared with optimized parameters by means of DFT calculations.

## 1. Introduction

**Figure 1:**
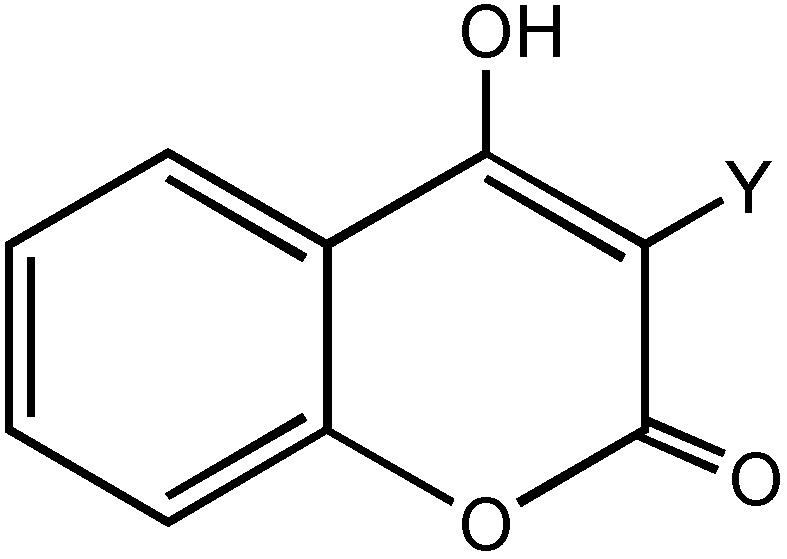
4-Hydroxy-3-substituted coumarins.

The 4-hydroxy-3-substituted coumarin moiety (Figure 1) is a common fused heterocyclic nucleus found in many natural products of medicinal importance. Several of these natural products exhibit exceptional biological and pharmacological activities such as antibiotic, antiviral, anti-HIV, anticoagulant and cytotoxicity properties [1,2,3,4,5,6,7,8]. Additionally, coumarin derivatives have been used as food additives, perfumes, cosmetics, dyes and herbicides [9,10]. Recently, Supuran *et al.* reported that coumarin derivatives constituted a totally new class of inhibitors of the zinc metalloenzyme carbonic anhydrase [11]. Additionally, two new series of 4-hydroxycoumarin analogues have been synthesized as inhibitors of the enzyme of human NAD(P)H quinine oxidoreductase-1 (NQO1), which is expressed in several types of tumor cells [12,13]. A series of coumarins bearing different groups on the aromatic ring were synthesized and tested as caspase activators and apoptosis inducers [14], showing that these compounds can be used to induce cell death in a variety of conditions in which uncontrolled growth and spread of abnormal cells occurs.

Moreover, coumarin dyes have attracted much interest owing to their application in organic light-emitting diodes (OLEDs). As a result of showing a wide range of size, shape and hydrophobicity, coumarins are used as sensitive fluorescent probes of systems including homogeneous solvents and mixtures and heterogeneous materials [15]. In addition, they form host-guest inclusion complexes with cage-like molecules such as cyclodextrins [16] and cucurbiturils [17].

The interest in the biological activity of 4-hydroxycoumarins continues nowadays, with warfarin and acenocoumarol being two of these derivatives which have been marketed as drugs [18,19]. Warfarin has been the mainstay of anticoagulation therapy worldwide for over 20 years, therefore a series of similar derivatives have been synthesized and tested as anticoagulant agents [20,21]. Acenocoumarol acts in the same way, therefore several 4-hydroxy coumarin derivatives have been synthesized and their pharmacological activity was tested [22,23,24,25,26]. 

**Figure 2:**
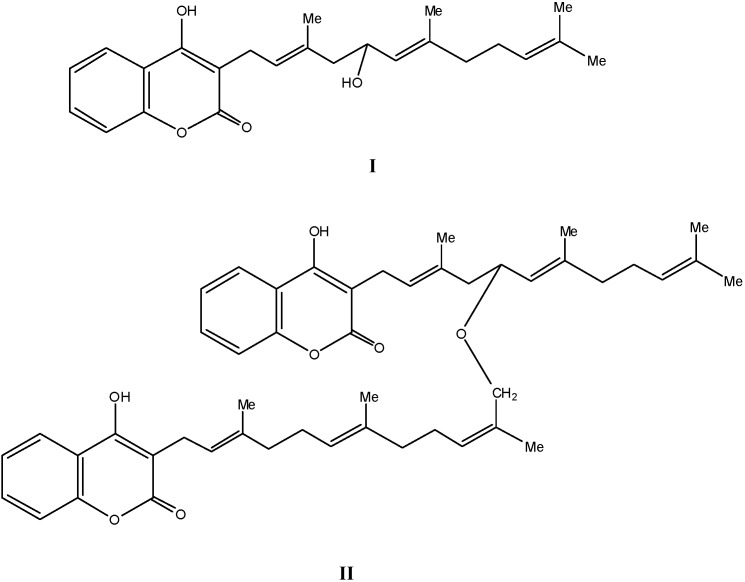
ε-Hydroxy ferulenol (**I**) and ferulenoxyferulenol (**II**).

**Figure 3:**
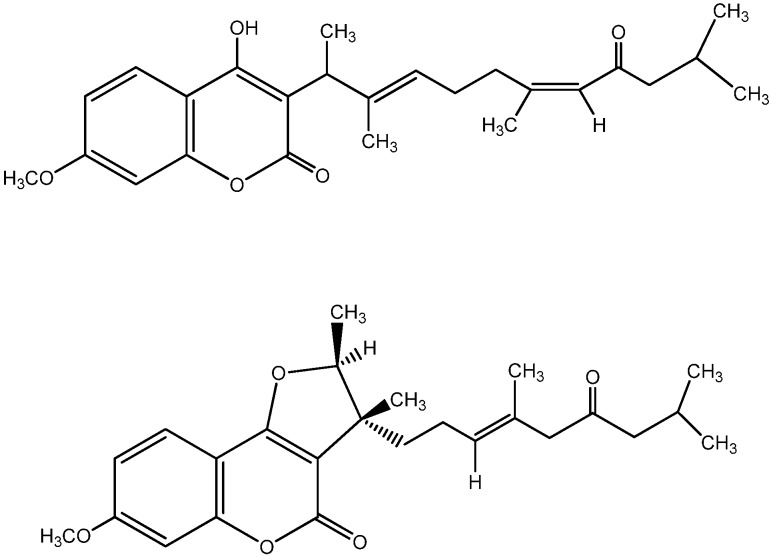
Sesquiterpenecoumarins.

A number of 4-hydroxy coumarins have been isolated from *Ferula* sp. The first ones were the toxic 3-fernesyl coumarin [27] and ferulenol [28] from *Ferula communis*. Many ferulenol derivatives followed [29,30,31,32,33] and the most recent ones are ε-hydroxy ferulenol (**I**) and ferulenoxyferulenol (**II**) (Figure 2). On the other hand, a number of sesquiterpenecoumarins have been isolated from *Ferulla pallid* [34]. Two new compounds (Figure 3) were isolated and their biosynthetic pathway was studied [35]. The synthesis of many compounds containing the 4-hydroxycoumarin nucleus showing antibacterial, insecticidal and activity against helminths has been reported [36]. A review article has been presented concerning the anti-HIV1 protease inhibition of a number of 4-hydroxycoumarins concluding that this inhibition is strongly dependent to the group attached at position 3 of the coumarin nucleus [37].

Coumarins and coumarin analogues have attracted the attention of many synthetic chemists since the late 1800s. Methods for their synthesis have been presented in the literature. Methodologies such as the Pechmann [38], Suzuki [8], Wittig [39] and Knoevenagel [40] condensation are well known. In addition, there have been reports for their synthesis using epoxides [41,42,43] or arylcarbamides as starting materials [44] or finally by intramolecular nucleophilic attack of β-ketoesters [45]. In this paper we used suitably functionalized salicylic acids as starting materials, as it has been reported in the literature [46,47].

## 2. Results and Discussion

**Scheme 1:**
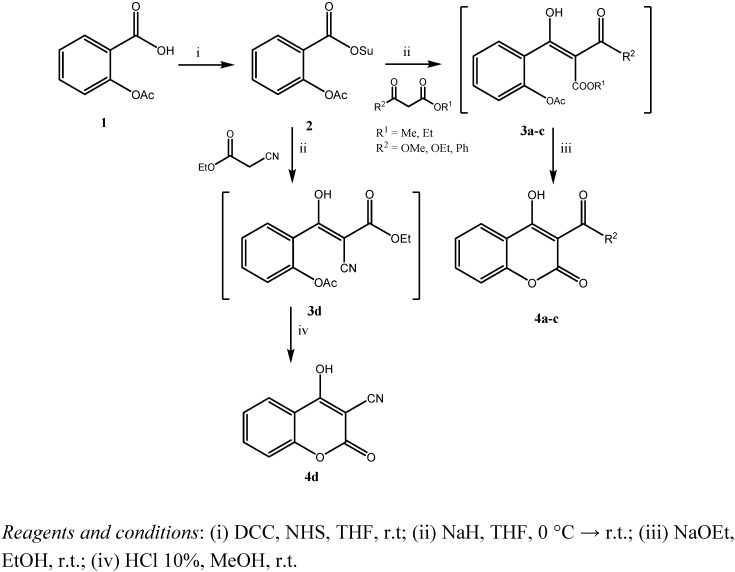
Synthesis of 3-functionalized-4-hydroxycoumarin-2-ones.

As part of our program studying the chemistry of fused heterocyclic systems with specific functional groups [47,48,49,50,51] we wish to report herein an extended methodology for the synthesis of 3-functionalized-4-hydroxycoumarin-2-ones, applying as alternative and ultimate scaffold, the *N*-hydroxysuccinimide ester of *O*-acetylsalicylic acid, for the “coupling reaction” with an active methylene compound. The chemistry proceeds via a tandem intermolecular nucleophilic coupling of the *N*-hydroxysuccinimide ester of *O*-acetylsalicylic acid **2** with an active methylene compound, and the subsequent intramolecular cyclization of the intermediate **3a-d** to a stable six-membered ring system, the coumarin nucleus **4a-d**, as shown in Scheme 1.

This approach would provide an alternative general method for the synthesis of coumarins and other similar organic molecules containing the benzopyranone ring system. The proposed protocol involves the following steps: a) the deprotonation of an active methylene compound; b) the nucleophilic attack at the carbonyl of the *N*-hydroxysuccinimide ester; c) the *in situ* intramolecular cyclization of the “intermediate” precursor affording the functionalized heterocycles bearing the coumarin nucleus. The key control element of this approach is the utilization of the *N*-hydroxy-succinimide ester of *O*-acetylsalicylic acid **2**. This acylating agent was synthesized by condensation of equimolar amounts of *O*-acetyl-protected salicylic acid **1** and *N*-hydroxysuccinimide (NHS) in the presence of 1.2 equiv. of dicyclohexylcarbodiimide (DCC) in anhydrous tetrahydrofuran at 0 °C. This excellent activating synthon **2** was isolated in good yields as a white solid and was used in the next step without further purification. The *C*-acylation protocol involved the reaction of 2 equiv. of an active methylene compound with 2 equiv. of sodium hydride in anhydrous tetrahydrofuran at 0 °C. After 1 hour of continuous stirring, 1 equiv. of the *N*-hydroxysuccinimide ester **2** was added and the mixture was stirred for 2 hours, at room temperature. In consequence, the solvent was removed under reduced pressure, the gummy solid was diluted with water, washed with diethyl ether and the aqueous layer was acidified with aq. solution of hydrochloric acid 10%, to give after extraction with dichloromethane, the intermediates **3a-d** as oily products. Cyclization of these *C*-acylation compounds was affected by refluxing them with two-fold excess amount of sodium ethoxide in ethanol for 24 h or by mixing them with aq. solution of hydrochloric acid 10% in methanol for 48 h at room temperature. 

Several features of the proposed methodology make it synthetically useful: the starting materials are inexpensive and stable; the yields are good; the reactions are relatively rapid and proceed at ambient temperature or under mild and easily controlled conditions. Furthermore, the methodology can be expanded to other heterocyclic systems bearing different heteroatoms or functions on the heterocyclic and/or aromatic ring.

### 2.1. X-ray Crystallographic Analysis

The crystal of this compound belongs to the monocyclic space group P2(1)/c. The data were collected at 150(2) K on a Bruker Apex II CCD diffractometer using Mo*K*_α_ radiation (λ = 0.71073 Å). The structure was solved by direct methods and refined on F^2^ using all the reflections [52]. Parameters for data collection and refinement are summarized in Table 1. 

**Table 1:** Crystal data and structure refinement for 4-hydroxy-3-methoxycarbonyl coumarin.

Empirical formula	C_11_H_8_O_5_
Formula weight	220.17
Temperature	150(2) K
Wavelength	0.71073 Å
Crystal system	Monoclinic
Space group	P2(1)/c
Unit cell dimensions	a = 3.802(3) Å
	b = 21.945(15) Å; β= 90.097(10)°.
	c = 11.352(8) Å
Volume	947.1(11) Å^3^
Z	4
Density (calculated)	1.544 Mg/m^3^
Absorption coefficient	0.124 mm^−1^
F(000)	456
Crystal size	0.44 × 0.10 × 0.07 mm^3^
Crystal description	colourless block
Theta range for data collection	0.93 to 25.00°.
Index ranges	−4 ≤ h ≤ 4, −25 ≤ k ≤ 26, −13 ≤ l ≤ 13
Reflections collected	7326
Independent reflections	1686 [R_int_ = 0.0758]
Completeness to theta = 25.00°	100.0%
Absorption correction	Semi-empirical from equivalents
Max. and min. transmission	0.9914 and 0.9474
Refinement method	Full-matrix least-squares on F^2^
Data / restraints / parameters	1686 / 0 / 149
Goodness-of-fit on F^2^	1.028
Final R indices [I > 2sigma(I)]	R_1_ = 0.0662, w_R2_ = 0.1633
R indices (all data)	R_1_ = 0.0984, w_R2_ = 0.1897
Largest diff. peak and hole	0.348 and -0.399 × 10^−3^ Å

**Figure 4:**
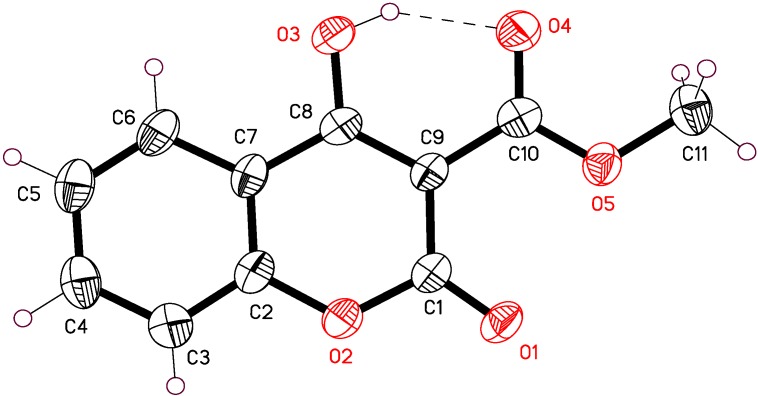
X-ray structure and numbering scheme of compound **4a**.

**Figure 5:**
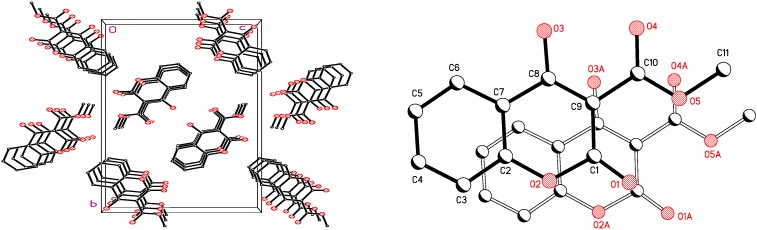
Packing diagram of 4-hydroxy-3-methoxycarbonyl coumarin **4a.**

Crystallographic data of 4-hydroxy-3-methoxycarbonyl- coumarin **4a** and selected bond lengths and angles are given in Table 2 and Table 3. The crystal structure and packing diagram of this compound are given in Figure 4 and Figure 5 respectively. 

**Scheme 2:**
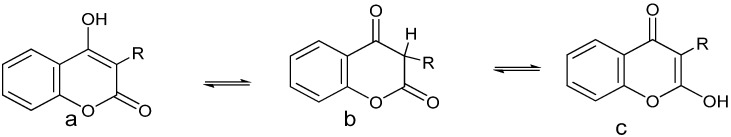
Tautomeric forms of 3-substituted-4-hydroxy coumarin.

The structure resembles that of tautomer a (Scheme 2) with a double bond character in C(8)-C(9) (1.37 Å) (Figure 4) and the bond C(8)-O(3) distinctly longer than the conventional carbonyl distance for C(1)-O(1) (1.31 Å and 1.19 Å respectively). The molecules show π-π stacking principally with a planar distance of 3.9 Å. Figure 5 shows this weak intermolecular π-π stacking interactions between molecules in crystal lattice. 

**Table 2:** Bond lengths [Å] and angles [°] for 3-methoxy-4-hydroxy coumarin.

C(1)-O(1)	1.199(4)	O(2)-C(2)-C(3)	116.7(4)
C(1)-O(2)	1.378(5)	O(2)-C(2)-C(7)	121.9(4)
C(1)-C(9)	1.450(5)	C(3)-C(2)-C(7)	121.4(4)
O(2)-C(2)	1.371(5)	C(2)-C(3)-C(4)	118.9(4)
C(2)-C(3)	1.374(6)	C(3)-C(4)-C(5)	120.6(4)
C(2)-C(7)	1.384(6)	C(6)-C(5)-C(4)	120.5(4)
C(3)-C(4)	1.376(6)	C(5)-C(6)-C(7)	119.3(4)
C(4)-C(5)	1.387(6)	C(2)-C(7)-C(6)	119.3(4)
C(5)-C(6)	1.372(6)	C(2)-C(7)-C(8)	117.3(3)
C(6)-C(7)	1.398(6)	C(6)-C(7)-C(8)	123.4(4)
C(7)-C(8)	1.435(6)	O(3)-C(8)-C(9)	123.3(4)
C(8)-O(3)	1.310(5)	O(3)-C(8)-C(7)	115.6(3)
C(8)-C(9)	1.375(5)	C(9)-C(8)-C(7)	121.1(3)
C(9)-C(10)	1.457(6)	C(8)-C(9)-C(1)	120.0(3)
C(10)-O(4)	1.232(5)	C(8)-C(9)-C(10)	118.3(3)
C(10)-O(5)	1.317(5)	C(1)-C(9)-C(10)	121.7(3)
O(5)-C(11)	1.441(5)	O(4)-C(10)-O(5)	121.8(4)
O(1)-C(1)-O(2)	115.0(3)	O(4)-C(10)-C(9)	121.9(4)
O(1)-C(1)-C(9)	127.8(4)	O(5)-C(10)-C(9)	116.3(3)
O(2)-C(1)-C(9)	117.2(3)	C(10)-O(5)-C(11)	116.4(3)
C(2)-O(2)-C(1)	122.4(3)		

**Table 3:** Hydrogen bonds for 4-hydroxy-3-methoxycarbonyl coumarin [Å and °].

D-H...A	d(D-H)	d(H...A)	D(D...A)	<(DHA)
O(3)-H(3A)...O(4)	0.84	1.77	2.512(4)	146.6

### 2.2. Quantum Chemical Calculations

The structure of 3-substituted-4-hydroxy coumarin consists of a benzene ring fused with a pyrone ring. The carbonyl group is attached at C-2 position, substitution group in position 3 and hydroxyl group in position 4. Three major tautomeric forms of 3-substituted-4-hydroxy coumarin can be formed and are presented in the following scheme (Scheme 2) [53,54].

Various quantum chemical calculations for coumarins have been previously reported in literature [55,56,57,58,59]. In order to predict the equilibrium molecular geometries of the possible tautomers of 3-methoxycarbonyl-4-hydroxy coumarin we have used the Density Functional Theory (DFT) hybrid method with the Becke’s three-parameter exchange functional and gradient-corrected functional of Lee, Yang and Parr (B3LYP) [60,61,62]. 

The geometries were fully optimized, with tight convergence criteria (Opt = Tight) using the following standard basis set: 6–311++G(d,p), valence triple zeta plus diffuse and polarization functions of d and p type. It is generally recognized that for an accurate description of hydrogen bonds at least double zeta quality basis augmented with a set of polarization and diffuse functions set is needed. Therefore a somewhat better geometry description is expected with 6–311++G(d,p) standard basis set. 

All geometry optimization were followed by calculations of frequencies in order to identify obtained structures as energy minima (no imaginary frequencies). All minima for the three tautomeric forms were verified by establishing that the matrix of energy second derivatives (Hessian) has only positive eigenvalues (all vibrational frequencies real). HOMO and LUMO frontier orbitals of the molecule were also computed at the same level of theory. All calculations were carried out with the Gaussian 09W program [63].

### 2.3. Computational Studies

**Figure 6:**
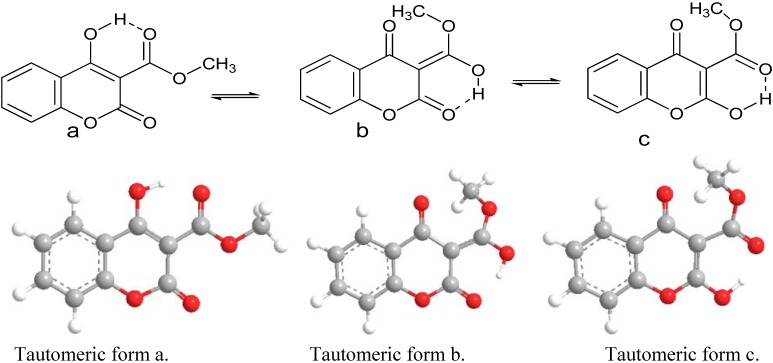
Optimized structures for the tautomers of 3-methoxycarbonyl-4-hydroxy coumarin using DFT.

DFT calculations have been performed for the tautomers of 3-methoxycarbonyl-4-hydroxy coumarin. Optimized molecular structures of the most stable tautomers are depicted in Figure 6. Their calculated energies and relative energies are presented in Table 4.

**Table 4:** Total Energy (a.u.) and relative energy for the four tautomers.

	Total Energy (kcal/mol)	ΔΕ
Tautomer a	502236.25	0
Tautomer b	502222.04	14.21
Tautomer c	502221.33	14.92

The optimized structure of compound **4a** which expected to be the predominant structure is shown in Figure 6 (Tautomer a) and it is close to the crystal structure given in the crystallographic analysis. As listed in Table 2 for selected bond and angles, it was found that the B3LYP/6–311++G(d,p) optimized structure is in good agreement with the X-ray crystallographic data as listed in Table 5. Therefore, the results using density functional theory (DFT) B3LYP/6–311++G(d,p) level is creditable. The average discrepancy of the selected bond lengths between theoretical and experimental data is less than ±0.02 Å and the average discrepancy of the selected bond angles is less than ±1.1°. 

**Table 5:** Comparison of bond lengths and angles.

**Bonds (Å)**	**X-ray**	**DFT**
C(1)-O(1)	1.199	1.198
C(8)-O(3)	1.310	1.319
C(10)-O(4)	1.232	1.238
O(2)-C(2)	1.371	1.356
C(10)-O(5)	1.317	1.323
C(1)-C(9)	1.450	1.463
C(9)-C(10)	1.457	1.469
C(8)-C(9)	1.375	1.394
**Angles (°)**	**X-ray**	**DFT**
O(1)-C(1)-O(2)	115	115.9
C(9)-C(10)-O(5)	116.3	116.3
C(10)-O(5)-C(11)	116.3	116.6

The predominant tautomer a is coplanar. Major variation in geometry of different tautomeric forms are at C8-O3, C9-C10, C10-O4, C1-O1, O5-C11, C10-O5 bonds and hydrogen bonds at O1, O3 and O5 atoms (O1-H, O3-H, O5-H). The remainder of the bonds and angles in the three tautomeric forms do not change significantly. Bond distances in the three tautomers indicate a more double bond character or a more single bond character as shown in Figure 6. According to the single-crystal structure distance shown in Table 2 predominance of tautomer a is confirmed. 

**Figure 7:**
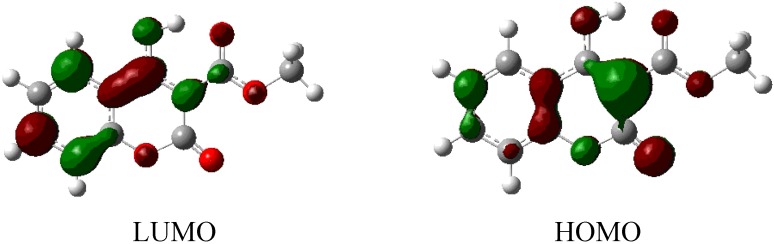
HOMO and LUMO orbitals of 3-methoxycarbonyl-4-hydroxy coumarin.

Molecular orbital calculations provide a detailed description of orbitals including spatial characteristics, nodal patterns and individual atom contributions. The contour plots of the frontier orbitals for the ground state of 3-methoxycarbonyl-4-hydroxy coumarin are shown in Figure 7 including the Highest Occupied Molecular Orbital (HOMO) and the Lowest Unoccupied Molecular Orbital (LUMO). It is interesting to see that both orbitals are substantially distributed over the conjugation plane. The HOMO and LUMO orbitals resemble those obtained for unsubstituted coumarin and therefore the substitution has only a small impact in the present case [64].

The orbital energy levels of HOMO, second HOMO (HOMO-1), LUMO and second LUMO (LUMO+1) of 3-methoxy-4-hydroxy coumarin were deduced using the DFT/6–311++G(d,p) method and are presented in Table 6. It can be seen that HOMO and LUMO energies are −0.25767 eV and −0.09207 eV respectively. The energy gap between HOMO and LUMO is about 0.1656 eV. 

**Table 6:** Orbital Energies.

Orbital	Energy (eV)
LUMO+3	−0.01268
LUMO+2	−0.01715
LUMO+1	−0.03766
LUMO	−0.09207
HOMO	−0.25767
HOMO-1	−0.27303
HOMO-2	−0.28980
HOMO-3	−0.30505

**Table 7:** Total Energy and relative energy for the four tautomers after single point PCM calculations.

	Total Energy (kcal/mol)	ΔΕ
Tautomer a	502251.36	0
Tautomer b	502236.36	15.00
Tautomer c	502236.08	15.28

**Figure 8:**
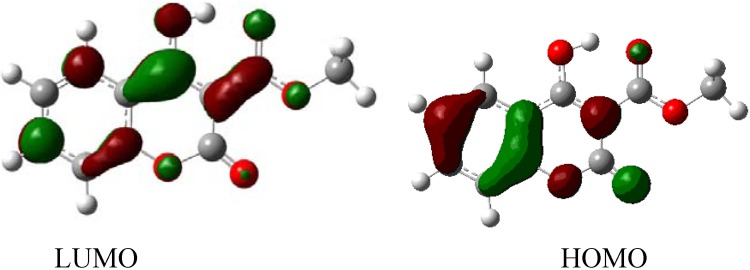
HOMO (−0.25988eV) and LUMO (−0.09171eV) orbitals of 3-methoxycarbonyl-4-hydroxy coumarin after single point PCM calculations.

On top of our gas phase geometries we have also performed single-point C-PCM (Conductor like Polarized Continuum Model) calculations using CH_2_Cl_2_ as the solvent. Results for relative energies and HOMO and LUMO orbitals are presented below in Table 7 and Figure 8 respectively. 

## 3. Experimental

### 3.1. General

All reagents were purchased from Aldrich, Fluka and Acros and used without further purification. Dry THF was distilled from Na/Ph_2_CO. Melting points were determined on a Gallenkamp MFB-595 melting point apparatus and are uncorrected. IR spectra were recorded on a Jasco 4200 FTIR spectrometer. NMR spectra were recorded on a Varian Gemini-2000 300 MHz spectrometer operating at 300 MHz (1H) and 75 MHz (13C). Chemical shifts δ are reported in ppm relative to DMSO-d_6_ (^1^H: δ = 2.50, ^13^C: δ = 39.52) and CDCl_3_ (^1^H: δ = 7.26, ^13^C: δ = 77.16). *J* values are given in Hz. 

#### 3.1.1. General Procedure for the Synthesis of the *N*-hydroxysuccinimide Ester of O-acetylsalicylic Acid (**2**)

*O*-acetylsalcylic acid (1, 10 mmol, 1.8 g) was treated under argon with *N*-hydroxysuccininimide (10 mmol, 1.16 g) in anhydrous THF (11.5 mL), and a solution of DCC (12 mmol, 2.47 g) in anhydrous THF (8.5 mL) was added dropwise at 0 °C. The reaction mixture was allowed to stir at 0 °C for 2 h. The resulting suspension was refrigerated overnight at 3-5 °C. The precipitated solid (DCCU) was filtered off and the filtrate was evaporated under reduced pressure and dried in vacuo to afford the *N*-hydroxysuccinimide ester of the corresponding O-acetylsalicylic acid as a white solid. Yield 2.4 g, 87%, mp 80 °C (lit. mp 87-89 °C [65]). ^1^H-NMR (DMSO-*d*_6_): *δ* 2.28 (s, 3H, COC*H_3_*), 2.88 (s, 4H, COC*H_2_*C*H_2_*CO), 7.39-7.42 (d, 1H, aromatic protons), 7.54 (pt, 1H, aromatic protons), 7.85 (pt, 1H, aromatic protons), 8.07-8.11 (d, 1H, aromatic protons). 

#### 3.1.2. General Procedure for the Synthesis of 3-Substituted-4-Hydroxycoumarins

Sodium hydride (60% in oil, 20 mmol, 0.8 g) was added in anhydrous THF (65 mL) at 0 °C and the resulting mixture was stirred under argon for 15 min at room temperature. The appropriate active methylene compound (ethyl benzoylacetate, diethyl malonate, dimethyl malonate, ethyl cyanoacetate (20 mmol) was then added at 0 °C, and the resulting mixture was stirred at room temperature for 1h. The *N*-hydroxysuccinimide ester of acetylsalicylic acid **2** (10 mmol) was added at 0 °C and the reaction mixture was stirred at r.t for 2h and then concentrated *in vacuo*. The obtained gum was diluted with H_2_O (10 mL) and washed with Et_2_O (10 mL). The aqueous extract was acidified with aqueous HCl (10%) in an ice-water bath to afford an oily product, which was extracted with CH_2_Cl_2_ (3 × 15 mL). The combined organic layers were dried over anhydrous Na_2_SO_4_, concentrated under reduced pressure and dried in vacuo to give the oily residue, which was treated either with method A or B.

Method A: The *C*-acylation compound (10 mmol) was added to a solution of sodium (0.46 g, 20 mmol) in absolute ethanol and stirred at room temperature for 24 h. The solvent was evaporated in vacuo and the residue was diluted with H_2_O and washed with Et_2_O, and the aqueous layer was acidified with 10% HCl at 0 °C to afford the desired coumarins as solid products. 

Method B: The C-acylation compound (10 mmol) was dissolved in MeOH (20 mL) and treated with aqueous HCl (10%, 20 mL) for 48 h at room temperature to afford a gummy solid which was extracted with CH_2_Cl_2_ (3 × 15 mL). The combined organic layers were dried with Na_2_SO4, concentrated under reduced pressure and dried in vacuo to afford the desired coumarins as solid products.

*4-Hydroxy-3-methoxycarbonylcoumarin* (**4a**): According to method A. White solid (1.41 mg, 64%), mp: 139-140 °C (lit. mp 139-140 °C [47]), IR (KBr) 1730,1640 (C=O), 1615 (C=C) cm^−1^; ^1^H-NMR (CDCl_3_): *δ* 4.02 (s, 3H, COOCH_3_), 7.30-7.38 (pt and dd, 2H, H-6, H-8), 7.65 (pt *J* = 8.1 Hz, 1H, H-7), 8.00 (d *J* = 8.1 Hz, 1H, H-5), 14.55 (s, 1H, OH); ^13^C-NMR (CDCl_3_): *δ* 53.1 (COO*C*H_3_), 93.2 (C-3), 114.6 (C-4a), 117.1 (C-8), 124.4 (C-6), 125.0 (C-5), 135.8 (C-7), 154.5 (C-8a), 157.6 (C-2), 172.5 (C-4), 175.7 (*C*OOCH_3_).

*3-Ethoxycarbonyl-4-hydroxycoumarin* (**4b**): According to method A. White solid (1.23 mg, 53%), mp 98.5-100 °C (lit. mp 100-101 °C [47]), IR (KBr) 1730,1638 (C=O), 1616 (C=C) cm^−1^; ^1^H-NMR (CDCl_3_): *δ* 1.43 (t *J* = 6.9 Hz, 3H, COOCH_2_C*H*_3_), 4.50 (q *J* = 6.9 Hz, 2H, COOC*H*_2_CH_3_), 7.28-7.36 (pt and dd, 2H, H-6, H-8), 7.67 (pt *J* = 8.1 Hz, 1H, H-7), 8.00 (dd *J* = 8.1/1.8 Hz, 1H, H-5), 14.73 (s, 1H, OH); ^13^C-NMR (CDCl_3_): *δ* 14.1 (COOCH_2_*C*H_3_), 62.9 (COO*C*H_2_CH_3_), 93.0 (C-3), 114.5 (C-4a), 116.8 (C-8), 124.2 (C-6), 125.0 (C-5), 135.5 (C-7), 154.2 (C-8a), 157.4 (C-2), 172.0 (C-4), 175.5 (*C*OOCH_2_CH_3_).

*3-Benzoyl-4-hydroxycoumarin* (**4c**): According to method A. White solid (1.83 mg, 69%), mp 115-116 °C (lit. mp 116-117 °C [47]), IR (KBr) 1722 (C=O), 1614 (C=C) cm^−1^; ^1^H-NMR (CDCl_3_): *δ* 7.30-7.70 (m, 8H, aromatic ring protons), 8.10 (dd *J* = 7.8/1.2 Hz, 1H, H-5), 16.72 (s, 1H, OH); ^13^C-NMR (CDCl_3_): *δ* 100.5 (C-3), 115.3 (C-4a), 117.4 (C-8), 124.6 (C-6), 125.7 (C-5), 128.1 (C-c), 128.5 (C-b), 132.7 (C-d), 136.3 (C-7), 137.8 (C-a), 155.2 (C-8a), 159.7 (C-2), 178.2 (C-4), 201.0 (COPh).

*3-Cyano-4-hydroxycoumarin* (**4d**): According to method B. White solid (0.60 mg, 35%), mp 252-254 °C (lit. mp 250-251 °C [47]), IR (KBr) 2247 (CN), 1717 (C=O), 1602 (C=C) cm^−1^; ^1^H-NMR (DMSO-*d*_6_): *δ* 7.23 (pt, 2H, H-6, H-8), 7.54 (pt *J* = 8.1 Hz, 1H, H-7), 7.81 (d *J* = 8.1 Hz, 1H, H-5); ^13^C-NMR (DMSO-*d*_6_): *δ* 75.0 (C-3), 116.3 (C-4a), 119.1 (C-8), 120.9 (CN), 123.0 (C-5), 124.9 (C-6), 132.6 (C-7), 153.5 (C-8a), 163.1 (C-2), 176.3 (C-4).

### 3.2. Crystal Structure Determination of 4a

Compound **4a**: C_11_H_8_O_5_, monoclinic, *P2* (1)/c, α = 3.802(3), b = 21.945(15), c = 11.352(8) A’, β= 90.097(10)°, V= 947,1(11) A’^3^, T=150(2) K, λ=0.71073 A’, Z = 4, 7326 reflections measured, 1686 unique (R_int_ = 0.0758), wR2 = 0.1897 (all data), R1 = 0.0662 (I > 2σ(Ι). Data were collected on a Bruker APEX II diffractometer. The structure was solved by direct methods and refined on F^2^ using all the reflections [60]. All the non-hydrogen atoms were refined using anisotropic atomic displacement parameters and hydrogen atoms bonded to carbon were inserted at calculated positions using a riding model. The hydrogen bonded to O3 was located from difference maps and refined with thermal parameter riding on that of the carrier atom. Crystallographic data (excluding structure factors) for the structure in this paper have been deposited with the Cambridge Crystallographic Data Centre as supplementary publication no CCDC 790977. 

## 4. Conclusions

In summary, we have successfully synthesized a range of functionalized 4-hydroxycoumarins using the *N*-hydroxysuccinimide ester of acetylsalicylic acid as a new efficient precursor (scaffold). The structure of 3-methoxy-4-hydroxy coumarin has been determined by single-crystal X-ray diffraction and its geometry was compared with optimized parameters obtained by means of Density Functional Theory calculations at B3LYP/6-311++(d,p) level. A good agreement between theory and X-ray diffraction was found. The HOMO and LUMO levels and the lowest energy tautomer of 3-methoxy-4-hydroxy coumarin have been studied with DFT at B3LYP/6–311++G(d,p) level. Further work in the benzopyranone series and the application of *N*-hydroxysuccinimide methodology towards the synthesis of more complex substrates with various substituents to explore potential biological applications will be reported in due course. 

## Supporting Information

**Table S1:** XYZ coordinates for tautomer a.

Center Number	Atomic Number	Atomic Type	Coordinates (Angstroms)		
			X	Y	Z
1	6	0	−3.863365	−1.026907	0.000004
2	6	0	−4.135989	0.349539	0.000006
3	6	0	−3.107716	1.279694	0.000004
4	6	0	−1.785738	0.829994	0.000001
5	6	0	−1.495749	−0.540269	−0.000001
6	6	0	−2.552628	−1.468517	0.000001
7	8	0	−0.806136	1.767112	−0.000002
8	6	0	0.568307	1.458477	−0.000005
9	6	0	0.902676	0.034388	−0.000002
10	6	0	−0.105367	−0.929393	−0.000003
11	8	0	0.138342	−2.225277	−0.000005
12	6	0	2.292934	−0.439924	−0.000002
13	8	0	3.231744	0.492372	0.000011
14	6	0	4.599130	0.032938	0.000012
15	8	0	1.315559	2.394467	−0.000013
16	8	0	2.573797	−1.645763	−0.000009
17	1	0	−4.677724	−1.740938	0.000006
18	1	0	−5.163665	0.694595	0.000009
19	1	0	−3.298328	2.345344	0.000005
20	1	0	−2.316874	−2.524743	0.000000
21	1	0	1.136344	−2.329785	−0.000006
22	1	0	5.197386	0.940375	0.000023
23	1	0	4.798717	−0.564118	−0.890153
24	1	0	4.798710	−0.564134	0.890168

**Table S2:** XYZ coordinates for tautomer b.

Center Number	Atomic Number	Atomic Type	Coordinates (Angstroms)		
			X	Y	Z
1	6	0	−3.618148	−1.245874	−0.007977
2	6	0	−3.998088	0.078140	0.245068
3	6	0	−3.047740	1.089629	0.295254
4	6	0	−1.709017	0.761144	0.096863
5	6	0	−1.304270	−0.549857	−0.147866
6	6	0	−2.281357	−1.553277	−0.207628
7	8	0	−0.810933	1.805230	0.121572
8	6	0	0.524803	1.604765	−0.018278
9	6	0	1.041859	0.251884	−0.085742
10	6	0	0.131388	−0.866082	−0.340040
11	8	0	0.488894	−1.980777	−0.710457
12	6	0	2.457879	0.140135	−0.089479
13	8	0	3.172725	−0.945992	0.051319
14	8	0	3.217744	1.187155	−0.227783
15	6	0	2.764139	−2.092598	0.836929
16	8	0	1.214329	2.624762	−0.071247
17	1	0	−4.368400	−2.026310	−0.049685
18	1	0	−5.042901	0.322058	0.399237
19	1	0	−3.318669	2.122060	0.476953
20	1	0	−1.954487	−2.565527	−0.411849
21	1	0	2.597225	1.992583	−0.271100
22	1	0	3.683895	−2.441204	1.303092
23	1	0	2.043747	−1.791336	1.597193
24	1	0	2.328808	−2.843394	0.186302

**Table S3:** XYZ coordinates for tautomer c.

Center Number	Atomic Number	Atomic Type	Coordinates (Angstroms)		
			X	Y	Z
1	6	0	3.625545	−1.219490	−0.028375
2	6	0	3.991918	0.105914	0.240048
3	6	0	3.030236	1.105326	0.304629
4	6	0	1.698956	0.755070	0.102571
5	6	0	1.303332	−0.553836	−0.159337
6	6	0	2.293164	−1.543919	−0.230248
7	8	0	0.776087	1.783523	0.141362
8	6	0	−0.522396	1.518830	0.014305
9	6	0	−1.048824	0.229110	−0.096703
10	6	0	−0.132598	−0.880766	−0.357093
11	8	0	−0.480906	−1.995785	−0.729157
12	6	0	−2.523855	0.155251	−0.147462
13	8	0	−3.193606	1.163428	−0.399711
14	8	0	−1.227759	2.608730	0.006259
15	8	0	−3.196434	−0.963313	0.091663
16	6	0	−2.723093	−2.031505	0.940363
17	1	0	4.385801	−1.989630	−0.080094
18	1	0	5.034104	0.359492	0.394986
19	1	0	3.287543	2.138811	0.499467
20	1	0	1.978969	−2.557818	−0.445769
21	1	0	−2.176000	2.299376	−0.206636
22	1	0	−3.600399	−2.352457	1.500562
23	1	0	−1.956518	−1.673761	1.628696
24	1	0	−2.326874	−2.836595	0.329283
